# Electrical Impedance Myography in Dogs With Degenerative Myelopathy

**DOI:** 10.3389/fvets.2022.874277

**Published:** 2022-05-27

**Authors:** Joseph B. Kowal, Sarah A. Verga, Sarbesh R. Pandeya, Randall J. Cochran, Julianna C. Sabol, Seward B. Rutkove, Joan R. Coates

**Affiliations:** ^1^Department of Veterinary Medicine and Surgery, University of Missouri, College of Veterinary Medicine, Columbia, MO, United States; ^2^Department of Neurology, Beth Israel Deaconess Medical Center, Harvard Medical School, Boston, MA, United States

**Keywords:** canine (dog), degenerative myelopathy, neuromuscular, atrophy, SOD1, electrical impedance myography (EIM)

## Abstract

Canine degenerative myelopathy (DM) leads to disuse and neurogenic muscle atrophy. Currently there is a lack of non-invasive quantitative measures of muscle health in dogs with DM. Muscle pathology has been previously quantified in other disorders using the technique of electrical impedance myography (EIM) but it has not been reported for DM. The objective of this study was to compare EIM between DM-affected and similar aged healthy dogs as well as assess EIM changes over time in DM-affected dogs. Multifrequency EIM was performed on DM affected dogs at baseline and during disease progression and on age-matched healthy dogs. Muscles evaluated in the pelvic limbs included the craniotibialis, gastrocnemius, gracilis, sartorius, and biceps femoris. The 100 kHz phase angle was extracted from the full frequency set for analysis. Phase values were lower in DM dogs as compared to healthy controls. Specifically, phase of the gastrocnemius was lower on the left (θ = 7.69, 13.06; *p* =0.002) and right (θ= 6.11, 11.72; *p* = 0.001) in DM vs. control dogs, respectively. The mean phase value of all measured muscles was also lower on the left (θ = 9.24, 11.62; *p* = 0.012) and right (θ = 9.18, 11.72; *p* = 0.021). Other individual muscles measured did not reach statistical significance, although values were consistently lower in DM-affected dogs. With disease progression, downward trends in phase values were detected in DM-affected dogs when monitored serially over time. This study demonstrates that EIM 100 kHz phase values are sensitive to muscle pathology in DM and that phase values are decreased in dogs with DM. Measurements from the gastrocnemius muscle show the greatest differences from similar aged healthy dogs suggesting it may be the preferred muscle for future EIM studies.

## Introduction

Degenerative myelopathy (DM) is a late adult-onset, progressive neurodegenerative condition in dogs that shares similarities with some forms of superoxide dismutase 1 (SOD1)—associated human amyotrophic lateral sclerosis (ALS) (1–4). Although initial signs reflect upper motor neuron deficits and general proprioceptive ataxia in the pelvic limbs, DM causes widespread degeneration of sensory and motor neuronal pathways in both the central and peripheral nervous system (5, 6). In dogs with early DM, muscles undergo disuse atrophy associated with the upper motor neuron paraparesis and a decrease in physical activity. If dogs are not euthanized in early disease stages, signs will progress to include flaccid tetraplegia and widespread muscle atrophy (4, 6–8). These lower motor neuron signs are a result of primary nerve fiber degeneration causing neurogenic muscle atrophy and associated secondary muscle pathology (6, 8–10). Specifically, histopathologic studies on pelvic limb and intercostal muscles of DM affected dogs showed atrophy, fibrosis, and variability in fiber diameter, type, and shape (6, 9). However, Pembroke Welsh Corgis in late disease showed uniformity in muscle atrophy (6). These findings suggested that pathology associated with DM may originate at least in part in the muscles such as related to sarcopenia. This conclusion is consistent with previously reported characterization of the thoracic intercostal muscles and nerves of DM affected PWCs in which muscle pathology was significant in the absence of any evidence of denervation (9, 10). Serial electrodiagnostic studies showed advancement of neuromuscular dysfunction by mild evidence of denervation on EMG, slowing of the motor nerve conduction velocity and a decrease in amplitude as well as temporal dispersion of the compound muscle action potential (6).

Objective disease biomarkers capable of enhancing diagnosis and tracking disease progression are needed to assist in development of therapies for canine DM. Various imaging studies, blood, cerebrospinal fluid, and electrodiagnostic biomarkers have been described for SOD-1 associated neurologic disease (11–15). Electrical impedance myography (EIM) is another tool that has the potential to evaluate individual muscles non-invasively and painlessly, making it possible to use in awake dogs (16, 17).

The general concept of EIM is that skeletal muscle can be modeled as a network of resistors and capacitors. When current is applied to muscle, resistance and capacitance change based on the physical size, integrity, and density of muscle fibers and alter the amplitude and timing of the voltage that is measured. The intracellular and extracellular matrices of muscle tissue cumulatively act as a resistor, and any atrophy that reduces the cross-sectional area of muscle tissue would be expected to increase the resistance (R); loss of both free extracellular and intracellular water will also contribute to increased resistance. The lipid bilayers that constitute muscle membranes act as capacitors, and as muscle atrophies, the local capacitance of the muscle increases (more membranes per unit volume of muscle). This capacitance is inversely proportional to reactance (X), and muscle atrophy, therefore, would be expected to decrease X. Impedance (Z) within biologic tissue circuits can be calculated using the equation *Z* = $\sqrt{R^{2}+X^{2}}$. Phase angle (or simply phase) (θ) combines R and X into a single value by the equation θ = arctan (X/R). It is the more commonly used outcome parameter, since it normalizes to some extent the anticipated simple volumetric impact of muscle loss. When a very high frequency electrical current is applied to a muscle and the resulting voltages measured, tissue reactance and resistance are obtained, and impedance and phase angle calculated. The integrity of individual cell membranes, myofiber size, and the presence of fat and connective tissue has a significant effect on the tissue's impedance; consequently, a muscle's impedance can be used to measure the tissue's alteration in disease progression. In a neurogenic disease, such as DM, resistance tends to increase and reactance tends to decrease, creating an even greater cumulative change in the phase value. Accordingly, impedance has been shown to change in a variety of neuromuscular conditions and can be used as an overall marker of muscle health (18).

Electrical impedance myography has been investigated as a non-invasive imaging biomarker in ALS patients (19–21), in children and a canine model with Duchenne muscular dystrophy (17, 22), and in a mouse model of ALS (23). Dystrophin-deficient dogs showed significant changes on EIM parameters of the biceps femoris muscle, with higher resistance and lower phase and reactance than normal dogs (17). Moderate strength correlations have been established between EIM and other neuromuscular biomarkers tracking ALS progression (21, 24, 25). In addition, phase values progressively decreased as disease progressed in ALS patients (20, 25).

The objectives for this project were to use EIM to characterize the location and extent of degenerative changes in the muscles of DM-affected dogs compared to similar aged healthy controls and to assess changes over time in dogs with DM. We hypothesize that DM-affected dogs would show lower phase angle values than healthy dogs, and that these values would show progressive decline in serially monitored DM-affected dogs.

## Materials and Methods

### Study Population

Healthy control and DM-affected dogs were prospectively recruited as part of various clinical trials being performed at the University of Missouri Veterinary Health Center. Since onset of DM is > 9 years of age, healthy dogs of similar ages were selected as controls to account for anticipated age-related changes in muscle. Control dogs were included if they were systemically healthy, had no history of neurologic or orthopedic disease, a normal complete blood count and serum biochemistry, a normal neurologic examination, and were not homozygous for the *SOD1*:c.118G>A genotype. DM-affected dogs were included if they were homozygous for *SOD1*:c.118G>A genotype, demonstrated clinical and neurologic signs compatible with DM, and had a thoracolumbar MRI with standard sequences excluding other myelopathies and supporting the presumptive diagnosis of DM. DM-affected dogs were participants in the following clinical trials: SOD1 epitope dimeric interface immunotherapy, canine SOD-1 antisense oligonucleotide trial, and an AAV.iSOD1 gene therapy trial with trial specific exclusion criteria. No treatment interventions were demonstrated to impact disease progression in any of the aforementioned clinical trials.

### Clinical Evaluation

Signalment and duration of neurologic signs, defined as the interval between the onset of gait deficits as described by the owner and the time of evaluation, were recorded for each dog with DM. Disease stage was determined using published criteria: stage 1, upper motor neuron paraparesis and general proprioceptive ataxia; stage 2, non-ambulatory paraparesis or paraplegia; stage 3, tetraparesis; stage 4, flaccid tetraplegia, widespread muscle atrophy and bulbar dysfunction (4). Dogs were considered early DM if in stage 1 or 2 and late DM if in stage 3 or 4. All DM-affected dogs and dogs in the healthy control population received physical and neurologic examinations prior to data collection.

### Electrical Impedance Myography Acquisition

**Figure 1:**
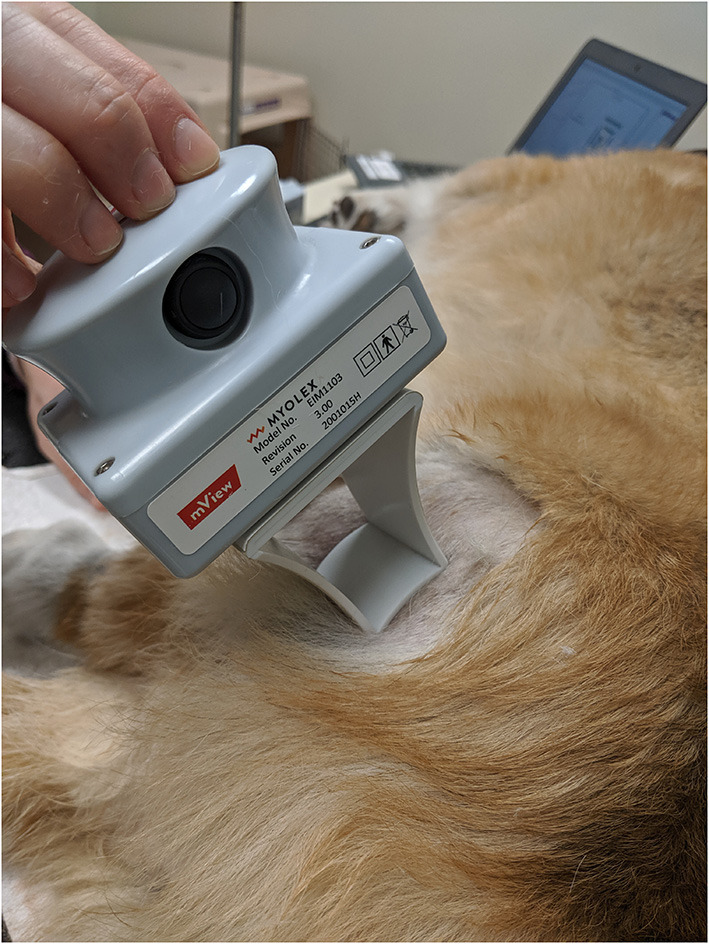
The handheld EIM recording device in place over the biceps femoris. Prior to recording, the fur was clipped and the site moistened with saline.

The handheld EIM mView device (Myolex, Inc, Boston, MA) was used to collect multi-frequency impedance data (Figure 1). The unit has a disposable multi-electrode array held in place *via* magnets and consists of three groups of electrodes, two nested sets for assessing current flow parallel to the major muscle fiber direction and one set for transverse measurement. The nested electrode design provides different depths of current penetration. Muscle selection was based on superficial location and ease of palpability. Selected muscles of the pelvic limbs evaluated included gastrocnemius, craniotibialis, gracilis, sartorius, and biceps femoris. The dog was awake throughout the assay and gently restrained in lateral recumbency. The probe was placed at the center of the bulk of the muscle (greatest width) for recordings. Prior to measurement, the fur on the skin overlying the muscles of interest was carefully removed with clippers to ensure a complete contact with the electrode. A saline soaked gauze sponge was applied to the skin for moistening to allow for conductance of current from the probe. The electrode surface of the EIM device was positioned over the identified muscle area on the skin as described in detail elsewhere (16). Values obtained included resistance (R), reactance (X), and phase (θ) at multiple frequencies of which 100 kHz was selected for analysis. Three recordings were obtained in quick succession from each muscle and recordings were repeated until repeatability between these three recordings was “good” or “excellent” based on automatic calculations in the provided Myolex^®^ software. When repeatability was “low” or “moderate,” the measurements were repeated up to five times before moving on to the next muscle. In some cases small changes to probe position were made in order to obtain repeatable measurements. The skin was remoistened with saline between each individual measurement. The five selected muscles were measured on both pelvic limbs in all DM-affected dogs and a portion of the healthy control dogs. Muscles evaluated in show Boxers, which constituted a portion of the healthy control dogs, excluded the left craniotibialis and biceps muscles because of clipping of fur in view of the judges was not permitted.

### EIM Data Processing

Prior to statistical analysis, raw data was subjected to specific sets of exclusion and inclusion criteria to eliminate influence of extreme observations and outliers, mainly due to poor electrode contact, which is easily identifiable on the multifrequency data obtained (showing major distortions at frequencies below about 20 kHz). Though EIM data was collected in multiple directions and frequencies, final analysis was performed only in the longitudinal direction (i.e., parallel to muscle fibers) using the outermost voltage electrodes at 100 kHz frequency. By using the outer electrodes, we ensured good muscle penetration and the 100 kHz frequency was chosen since it is generally unaffected by contact artifacts. While various multifrequency measures have been used in various other EIM studies and applications, given the preliminary nature of this work, we decided to focus on this single frequency leaving additional analysis for future work. The phase angle (θ), which has been previously established as a primary EIM outcome measure in neuromuscular disease and is generally unaffected by muscle size or shape alterations, was the primary outcome assessed here (18). In addition, individual muscle data points were removed as outliers from final analysis if outside of biologically possible ranges (negative values or >30°) or if they were >2 standard deviations away from the mean for that muscle. For DM affected dogs with EIM collection at multiple time points, only the first collection point (baseline) was used for comparison to the healthy control population.

### Statistical Analysis

Analysis was conducted in R 4.0.3 (R Foundation for Statistical Computing, Vienna, Austria). Normality was checked using the Kolmogorov-Smirnov Test and QQ plots. Based on normality assessment, a non-parametric approach (Wilcoxon rank sum test) was used to compare DM-affected dogs to age-matched controls. Linear trend plots were created to assess muscle deterioration in diseased dogs over time using a mixed model approach; the slope of the model, as indicated by β, was recorded as negative or positive. Negative β would therefore indicate progressive decreases in EIM phase over time. A mixed model approach was also used to compare the EIM changes over time with gait score in DM-affected dogs, specifically in the gastrocnemius muscle. Intraclass correlation coefficients (ICC) were calculated to assess reliability of repeated EIM measurements using the second and third recording from each muscle. Statistical significance was determined at *p*-value < 0.05, and all results are presented as mean ± standard deviation.

All tests were conducted for each of the five muscles on the left and right side. Additionally, an overall mean phase of all five muscles on both sides was determined in each dog.

## Results

### Study Population

Twenty-six dogs were included in the study, including 11 dogs presumptively diagnosed and later histopathologically confirmed with DM, and 15 healthy control dogs. Age-similar controls were selected (range 8–13 years) from a previously obtained larger group of normal dogs, resulting in a total of 15 dogs for analysis in the control group. Of the 11 dogs with DM, seven had multiple EIM recordings throughout disease progression and were used for longitudinal analysis. The mean age at baseline collection of the DM-affected group was 11.1 ± 3.0 years. The mean time (in months) from onset of clinical signs of DM in the affected group was 10.2 +/– 7.3 months. The sex distribution within the DM group included spayed female (*n* = 5), castrated male (*n* = 6), intact female (*n* = 1), and intact male (*n* = 1). Breeds included Boxer (*n* = 4), Pembroke Welsh Corgi (*n* = 4), mixed breed (*n* = 3), Soft Coated Wheaten Terrier (*n* = 1), and German Shepherd Dog (*n* = 1). All dogs in the DM affected group were homozygous for the *SOD1c.118A* allele. During the first EIM recording, nine dogs in early disease stage were ambulatory paraparetic and two dogs were non-ambulatory paraparetic or paraplegic.

The mean age of the control group was 10.1 ± 1.8 years. Sex distribution included: spayed female (*n* = 5), castrated male (*n* = 8), and intact male (*n* = 2). Breeds included Boxer (*n* = 7), mixed breed (*n* = 2), and one each of the following breeds: Beagle, German Shepherd Dog, Portuguese Water Dog, Treeing Walker Coonhound, Rat Terrier, and Jack Russell Terrier. The SOD1 genotype was homozygous normal (G/G) in nine dogs and heterozygous (G/A) in six dogs. No dogs with a homozygous affected/at risk status were included.

### Evaluation of EIM Phase Values in DM-Affected and Control Dogs

**Figure 2:**
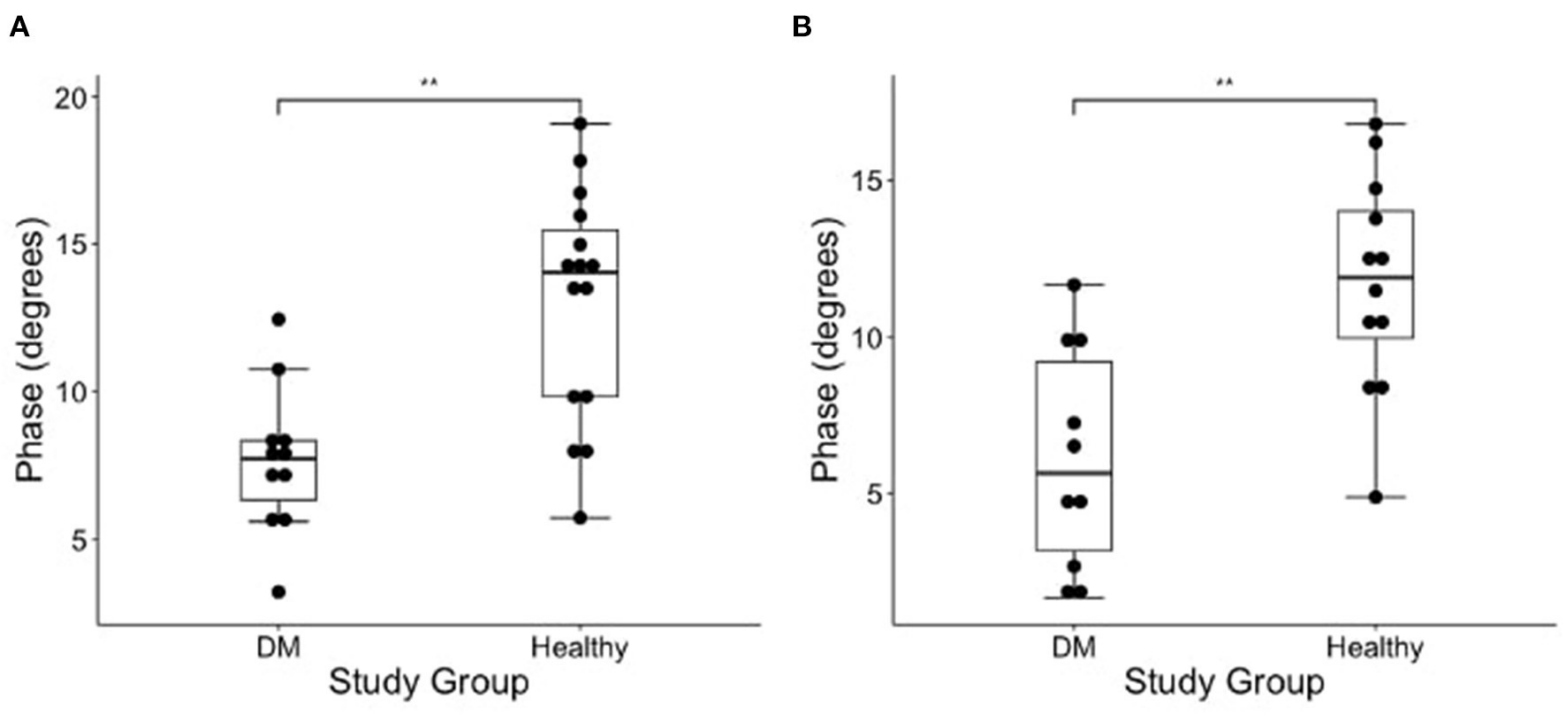
Boxplot of the EIM phase measurements (degrees) obtained from the **(A)** left and **(B)** right gastrocnemius muscles in DM dogs and healthy dogs using Wilcoxon rank sum test. There is a significantly lower phase in the gastrocnemius of the DM affected dogs as compared to healthy control dogs in both pelvic limbs [**indicates significance; *p*-value = 0.002 (left) and 0.001 (right)].

**Figure 3:**
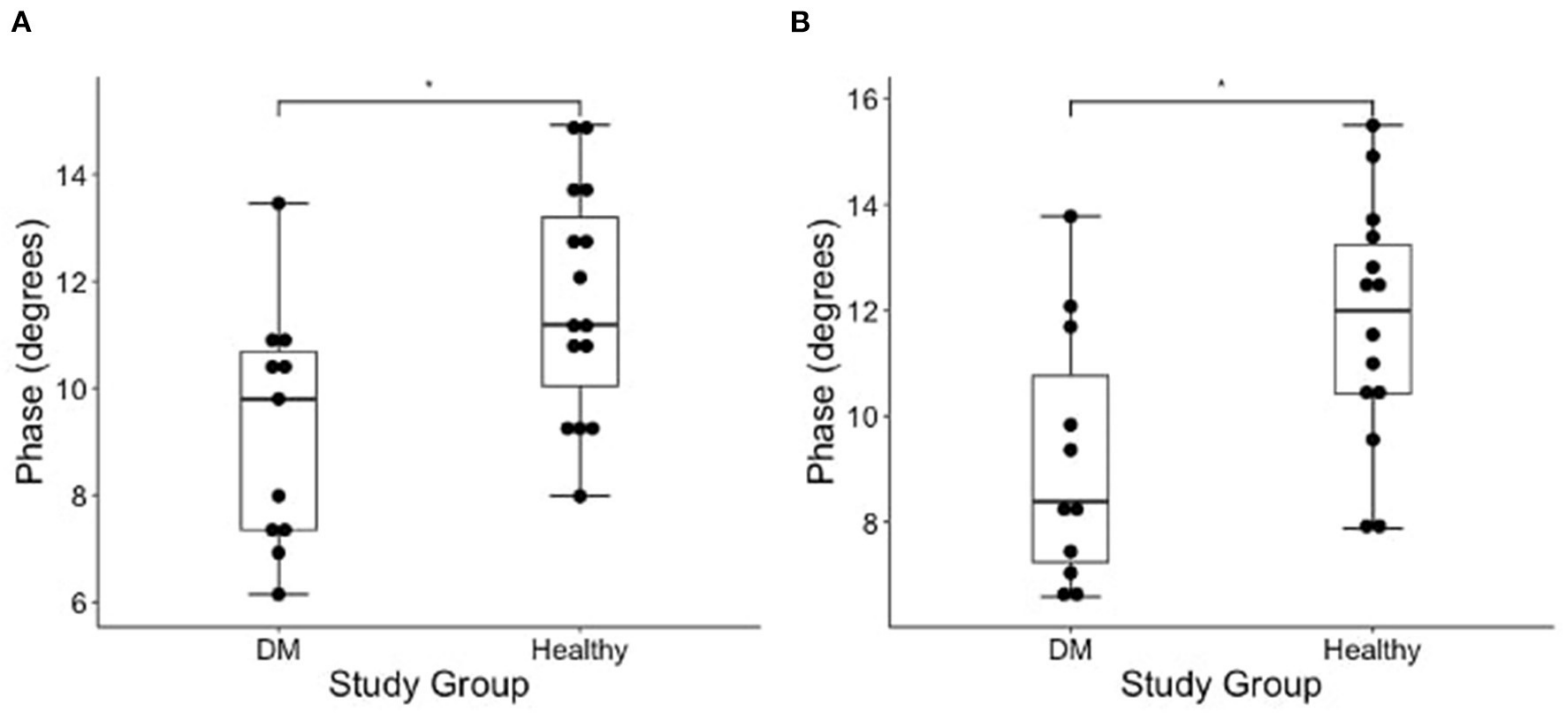
Boxplot of the EIM phase measurements (degrees) obtained from average of all measured pelvic limb muscles on the **(A)** left and **(B)** right side muscles in DM dogs and healthy dogs using Wilcoxon rank sum test. There is a significantly lower phase of the DM affected dogs as compared to healthy control dogs in both pelvic limbs [*indicates significance; *p*-value = 0.012 (left) and 0.021 (right)].

The results of EIM phase values in DM-affected and control dogs are summarized in Table 1. Compared with healthy dogs, mean phase value was decreased in the left and right gastrocnemius muscle of DM affected dogs (Figure 2; *p*-value = 0.002 and 0.001, respectively). No significant difference was detected in the craniotibialis, biceps femoris, gracilis, or sartorius. When average phase value from all muscles was combined, a significant difference was detected between DM affected and healthy dogs on the left and the right (Figure 3; *p*-value = 0.012 and 0.021, respectively).

**Table 1:** Phase values in DM-affected vs. control dogs and intraclass correlation coefficient of repeated phase measurements.

	**Muscle**	**Left**			**Right**		
		**Sample size (DM, healthy)**	**Mean (±Std) DM, healthy**	***p*** **-value**	**Sample size (DM, healthy)**	**Mean (±Std) DM, healthy**	***p*** **-value**
Difference of phase means (Wilcoxon rank sum test)	Craniotibialis	(10, 13)	12.01 (± 3.60); 13.81 (± 3.09)	0.186	(11, 13)	11.01 (± 2.88); 13.66 (± 3.57)	0.082
	Biceps femoris	(10, 12)	7.98 (± 4.35); 9.91 (± 4.14)	0.428	(10, 12)	9.33 (± 6.91); 10.28 (± 4.23)	0.417
	Gracilis	(11, 15)	7.86 (± 1.93); 9.75 (± 2.66)	0.053	(10, 14)	9.00 (± 4.03); 10.31 (± 3.89)	0.471
	Gastrocnemius	(11, 15)	7.69 (±2.48); 13.06 (±3.92)	0.002^*^	(10, 12)	6.11(± 3.54); 11.72 (± 3.48)	0.001^*^
	Sartorius	(11, 14)	10.51 (± 2.42); 11.39 (± 2.67)	0.403	(9, 14)	9.38 (± 3.32); 12.21 (± 2.88)	0.072
	Average	(11, 15)	9.24 (± 2.23); 11.62 (± 2.15)	0.012^*^	(11, 14)	9.18 (± 2.42); 11.72 (± 2.34)	0.021^*^
		**Sample size (DM, healthy)**	**ICC**		**Sample size (DM, healthy)**	**ICC**	
Intraclass correlation coefficient	Craniotibialis	(10, 13)	0.919		(11, 13)	0.939	
	Biceps femoris	(9, 12)	0.916		(9, 12)	0.918	
	Gracilis	(11, 15)	0.83		(10, 14)	0.94	
	Gastrocnemius	(11, 15)	0.943		(10, 11)	0.89	
	Sartorius	(11, 14)	0.861		(9, 14)	0.97	
	Average	(11, 15)	0.932		(11, 14)	0.924	

*ICC, Intraclass correlation coefficient*.

* *p-values < 0.05 indicate significance*.

*ICC values > 0.75 indicate good repeatability*.

### Changes in EIM Phase Over Time in DM-Affected Dogs

**Figure 4:**
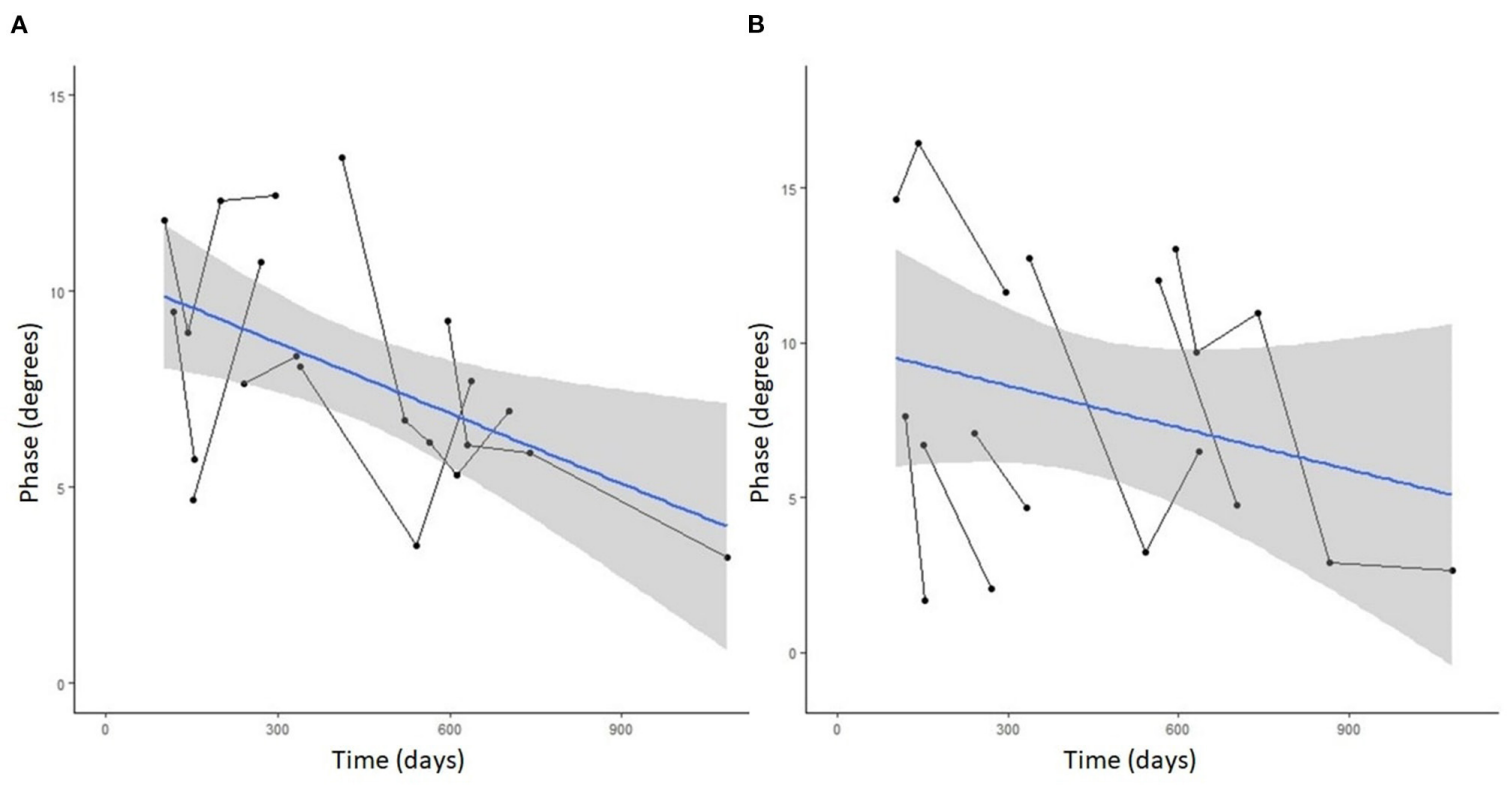
Spaghetti plot highlighting EIM phase changes over time in the **(A)** left and **(B)** right gastrocnemius muscle in serially monitored dogs with DM. The time in days is measured from the onset of clinical signs attributable to DM. There is a gradual reduction of the phase value in the gastrocnemius muscle over time in dogs with DM.

**Figure 5:**
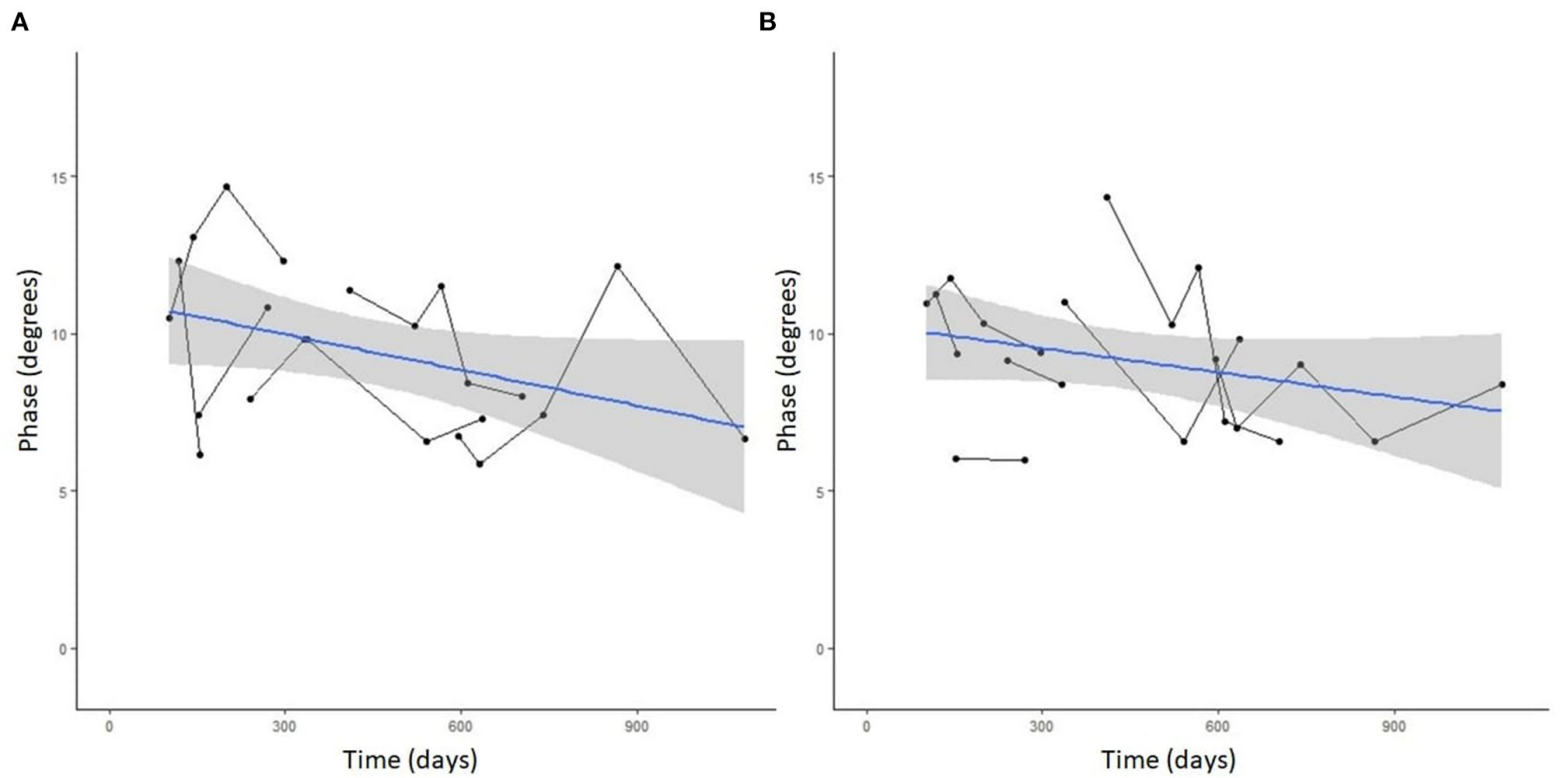
Spaghetti plot highlighting mean EIM phase changes across all muscles in the **(A)** left and **(B)** right pelvic limbs in serially monitored dogs with DM. The time in days is measured from the onset of clinical signs attributable to DM. Muscles included in means are the gastrocnemius, craniotibialis, biceps femoris, gracilis, and sartorius.

Seven DM-affected dogs had EIM data collected at multiple time points during disease progression (between 2 and 5 collections), which occurred at ~3 month intervals. The mean phase trend plots for all muscles over time among DM affected dogs showed a negative slope β (Table 2), except right gracilis which showed a positive slope; this reduction in time was significant for gastrocnemius [*p*-value 0.03 (left) and 0.0016 (right)]. The mean phase trend plot is shown for the gastrocnemius muscle (Figure 4) and the average of all muscles (Figure 5).

**Table 2:** Mixed model analysis of longitudinal progression in DM affected dogs.

**Muscle**	**Intercept**	**β (*p*-value)**	**Intercept**	**β (*p*-value)**
Craniotibialis	11.98	−0.002 (0.40)	12.30	−0.003 (0.44)
Biceps femoris	10.17	−0.003 (0.44)	10.62	−0.003 (0.62)
Gracilis	10.05	−0.004 (0.11)	7.72	0.001 (0.76)
Gastrocnemius	10.32	−0.0057 (0.03)^*^	14.99	−0.019 (0.0016)^*^
Sartorius	10.41	−0.0024 (0.40)	9.82	−0.0031 (0.35)
Average	10.67	−0.0031 (0.19)	11.48	−0.005 (0.15)

*Negative β-values indicate a negative trend and progressive decreases in the EIM phase value over time*.

* *p-value < 0.05 indicates significance*.

### Repeatability of EIM Measurements

**Figure 6:**
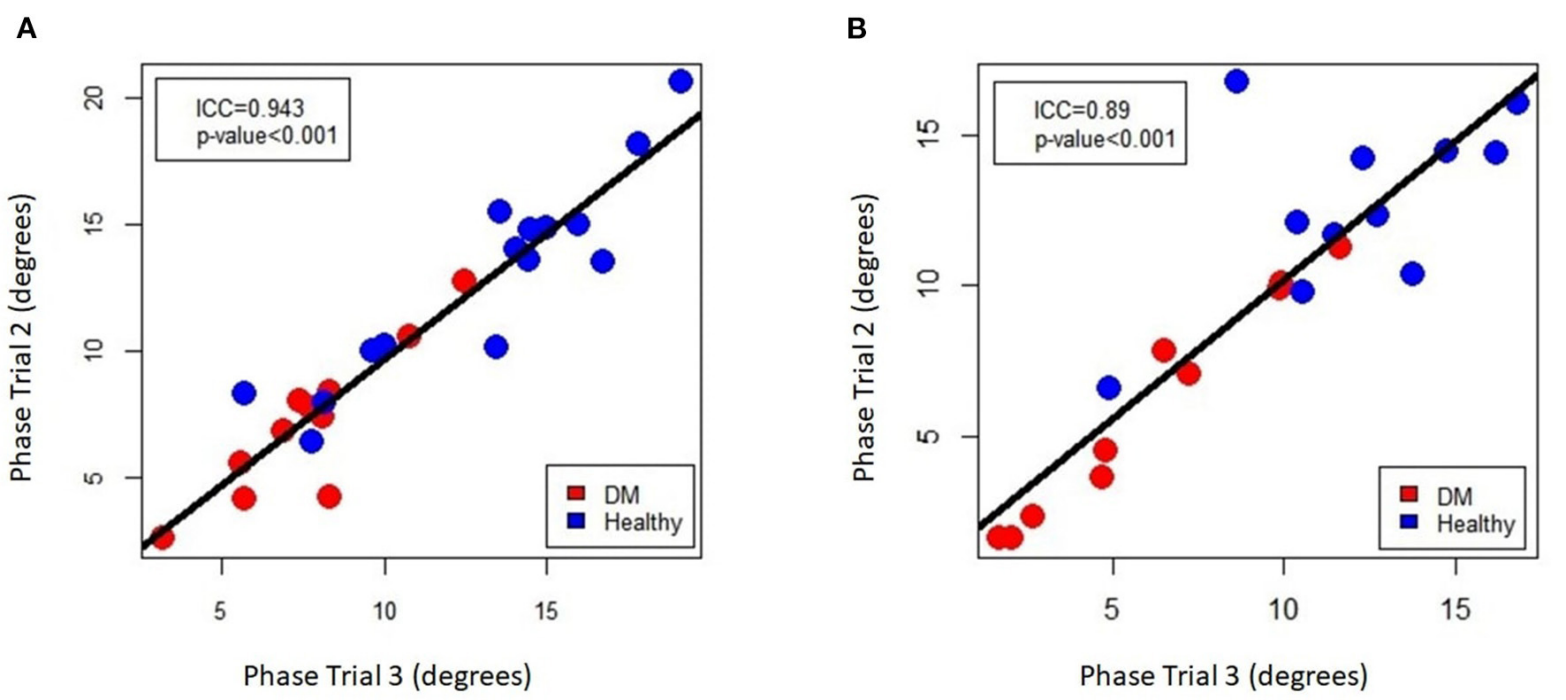
Intra-class correlation coefficient plot of EIM phase measurements for repeated sampling of the **(A)** left and **(B)** right gastrocnemius muscle, comparing the 2nd and 3rd of the three data acquisitions that were performed on each muscle.

To assess for repeatability of the EIM measurements the second and third trials during the first EIM recording from DM-affected dogs and control dogs were compared for each collection point *via* intraclass correlation coefficient (ICC). ICC was above 0.75 for all muscles (Table 1), indicating good repeatability. The ICC graph for the gastrocnemius muscles is shown in Figure 6.

### Relation of EIM Parameters With Gait Deficit Score and Time

To assess the impact of clinical progression of DM on the EIM parameters, mixed models were created with the EIM phase as a dependent variable and the gait score and time as independent variables for both the left and the right gastrocnemius muscles among the DM-affected dogs. No correlation between gait deficit score and phase was observed nor was there a significant interaction between time and gait score (all *p*-values all greater 0.1).

## Discussion

This study reports the first use of EIM as a measure for disease progression of canine DM. We demonstrate the utility of EIM to differentiate muscle alterations associated with DM compared to muscles in healthy control dogs. Additionally, EIM may be a useful measure for long-term monitoring of disease progression in individual dogs. Currently, only physical therapy has been shown to prolong survival time in dogs with DM, though this may be subject to owner bias with those owners willing to pursue physical rehabilitation being more likely to provide nursing care for dogs as the disease progresses (26). Regardless, EIM may be useful in monitoring muscle health in dogs undergoing physical rehabilitation.

Denervation muscle atrophy, such as that which occurs in dogs with late DM, results in pathologic changes which impact EIM phase. Biophysical changes to myofibers and connective tissue in an atrophic setting markedly alters the electrical properties of the tissue. Specifically, decrease in myofiber size and increase in fat and connective tissue deposition cause an increase in EIM resistance and a decrease in reactance values in certain frequency ranges. These changes to resistance and reactance cause the resulting phase angle to decrease as we observed here (18). In Boxers with DM, atrophic fibers with normal intramuscular nerve branching have been observed in the early stages of the disease (6). These changes are most consistent with the disuse atrophy seen in the early upper motor neuron stages of DM (4). In the late DM, in which dogs have chronic lower motor neuron signs, there are larger groups of atrophic fibers and extensive fiber-type grouping. In Pembroke Welsh Corgis, which are frequently maintained into late DM, diffuse muscle fiber atrophy has been observed without significant fiber type grouping. This has been hypothesized to be secondary to denervation with loss of reinnervation and accelerated sarcopenia (6). These histopathologic changes to muscle form the basis for the hypothesized changes in EIM phase with progression of disuse to neurogenic atrophy. In people with disuse atrophy, EIM on affected muscles show lower phase values when compared to normal muscle (27). Thus, EIM should be able to monitor muscles for DM progression in both the early and late stages of disease for disuse and neurogenic atrophy, respectively.

In the early stages of DM, pelvic limb deficits are asymmetric. Though in a random population of dogs with DM this variability would average out, we assessed the left and right pelvic limb muscles independently in this study. The reasoning for this is 2-fold. First, some of the healthy control Boxers were participating in show events and handlers would only allow clipping of hair on the limb that would be away from the judges, and thus EIM data could only be collected on one limb. Second, in human clinical trials for ALS, it is routine to assess limbs independently in case response to therapy and EIM measurements are asymmetric.

Muscles selected were based on easy identification, size large enough to accommodate the EIM probe, and proximal and more distal locations. The mean phase of the pelvic limb muscles in dogs with DM at the time of their first assessment was significantly lower compared to similar aged healthy dogs. A similar finding was also present when the gastrocnemius was assessed individually, but no significant differences were detected when other muscles were assessed on their own. The gastrocnemius muscle likely showed the most consistent changes between DM-affected and healthy dogs due to its more caudodistal location and potentially higher susceptibility to atrophy secondary to distal axonopathy. However, based on this reasoning, it was expected the craniotibialis muscle to have similar findings, but this was not the case. The gastrocnemius' larger size, separation from other muscles and tissues, less coverage by adipose, and general ease of measurement may have thus also played roles. The other evaluated muscles are generally smaller and the EIM probe placement overlies adjacent muscle and fascial tissues, which may impact phase values. The ease of palpating the gastrocnemius, its large size, and strong repeatability as demonstrated by ICC may make it a good candidate muscle for use in future studies as the sole muscle needed for longitudinal testing with EIM in DM.

The degree of muscle atrophy in dogs with DM is progressive over time and ranges from mild secondary to disuse in early stages to severe neurogenic atrophy in late stages of the disease (6). These histopathologic alterations would lead to progressive decreases in phase values as assessed at multiple time points in DM-affected dogs. The initial phase values and rate of progression varied in this cohort of dogs, indicating that phase alone should not be used as an initial diagnostic marker for the disease. However, trends over time in phase in the individual dog may be helpful in assessing progression and response to therapies.

A mixed model approach was also used to compare clinical progression (*via* the DM gait deficit score) to EIM phase values and time. A higher gait deficit score is associated with further progression of DM clinical signs. No correlation was observed, which suggests that the EIM phase is not useful for predicting disease stage alone. However, given the relatively small number of animals included in this analysis and considerable variation in the stage of disease and length of follow-up for the animals, this analysis was likely severely underpowered to detect such a relationship.

To assess for repeatability between repeated measurements of the same muscle, intraclass correlation assessments were performed. An ICC value of 0.75 or greater, generally indicating good repeatability, was obtained for all muscles. The overall strong ICC values of for all muscles indicates the ease of use of the device in obtaining repeatable measurements.

This study and that of Hakim et al. in 2017, which assessed EIM use in dogs with muscular dystrophy, support the use of EIM as diagnostic tool and biomarker for tracking disease progression in dogs with diseases causing muscle atrophy and dysfunction (17). The changes in phase over time documented in dogs with advancing signs of DM support its use alongside standard electrodiagnostic testing including nerve conduction studies and electromyography. Major benefits of EIM in longitudinal tracking, compared to other measures, include it being a painless and non-invasive procedure and not requiring anesthesia. Additionally, repeated placement of needles into small muscles, as required by EMG, may accelerate further damage to muscles already vulnerable to injury. Finally, collection of EIM data is rapid (20–30 s per muscle) and can be performed by any individual after only a few minutes of training and without specific knowledge of the underlying technology.

Several limitations should be considered when interpreting the results of this study. First, the study population of DM-affected dogs was relatively small and a power analysis was not performed. Additionally, other factors that may impact impedance measurements such as sex, body condition score, and muscle condition score were not assessed in this study. The location and severity of muscle atrophy was not recorded and histopathologic analysis of the studied muscles was not performed. This study was intended as a proof-of-concept for the use of EIM in dogs with DM; these other factors may be valuable to include in future studies. Thoracic limbs measurements were not performed, though in future studies including them as an internal control in early stages of disease may prove useful. Dogs were enrolled in various clinical trials evaluating novel treatments for DM. Although there was no effect of treatment on disease progression, the treatment could still affect the EIM data collected from DM-affected dogs. Data collection was performed by multiple individuals which may have led to subtle variation in electrode placement on individual muscles, though a standardized protocol was established to help minimize this effect. Due to the nature of clinical research and availability of personnel at various timepoints in the study, each cohort of dogs had one individual primarily collecting data for the group, making assessments of inter-rater reliability impossible in this study. Though not performed here, high inter-rater reliability when using a handheld EIM device has been substantiated elsewhere in humans (28). As muscle atrophy became more severe in DM affected dogs, probe placement over atrophied muscles became difficult and adjacent structures or other muscles may have been included in measurements due to the fixed size of the recording probe. However, there is nothing precluding the development of smaller, dog-sized probes in future work. An additional limitation of this study is the absence of any longitudinal data on the healthy dogs. We suspect that advancing age will impact EIM values in that group too, as sarcopenia develops, but not to the extent observed in the DM dogs; however, that will need to be the subject of future inquiries. Finally, a control group of dogs with other non-neurologic mobility impairments may have been helpful in determining the role of disuse atrophy in the observed phase changes. Unfortunately, the resources and patient population were not available to include such a cohort of dogs. The demonstration of decreased phase values in people with disuse atrophy secondary to placement of an immobilizing cast serves to demonstrate that phase changes are seen with both disuse and neurogenic atrophy (27).

As a pilot investigation for the use of EIM in DM, the focus of this study was purposefully narrow. Future work may focus on comparison of normal dogs of various breeds and body conditions, further refinement of muscle landmarks for data collection, additional analysis of compound multifrequency measures, and assessment of EIM changes over time in the aging animal. Comparison of impedance measurements in dogs affected with DM to the current standard of electrodiagnostic testing would also be warranted. Ultimately the use of EIM to track longitudinal progression of DM and response to novel therapies may be possible as is currently done for ALS trials in people. Other areas of veterinary medicine for applications of EIM could include physical rehabilitation, disease monitoring in orthopedic and neurologic diseases, nutritional studies, or assessment of muscle health in working or agility dogs.

In conclusion, this study demonstrates that EIM is a useful tool in detection of pathologic changes to muscle in DM-affected dogs. The EIM phase of affected muscles is lower in dogs with DM compared to healthy control dogs. Additionally, a trend in decreasing phase values is observed during longitudinal evaluation of dogs affected with DM. Future studies are necessary to solidify the utility of EIM as a tracking biomarker for DM and to determine its usefulness as a monitoring parameter in clinical trials.

## Data Availability Statement

The raw data supporting the conclusions of this article will be made available by the authors, without undue reservation.

## Ethics Statement

The animal study was reviewed and approved by University of Missouri Animal Care and Use Committee. Written informed consent was obtained from the owners for the participation of their animals in this study.

## Author Contributions

JK and JC contributed to the conception and design of the study. JK, JC, RC, and JS contributed to data collection. SR, SV, and SP performed the data analysis. JK wrote the first draft of the manuscript. All authors contributed to manuscript revision, read, and approved the submitted version.

## Funding

ALS Association TREAT ALS (15-LGCA-249) and University of Missouri, College of Veterinary Medicine Phi Zeta Grant Award both grants contributed to recruitment of cases and data collection.

## Conflict of Interest

SR has equity in, and serves a consultant and scientific advisor to Myolex, Inc. and Haystack Diagnostics, companies that design impedance devices for clinical and research use; he is also a member of the company's Board of Directors. The companies also have an option to license patented impedance technology of which SR is named as an inventor. The remaining authors declare that the research was conducted in the absence of any commercial or financial relationships that could be construed as a potential conflict of interest.

## Publisher's Note

All claims expressed in this article are solely those of the authors and do not necessarily represent those of their affiliated organizations, or those of the publisher, the editors and the reviewers. Any product that may be evaluated in this article, or claim that may be made by its manufacturer, is not guaranteed or endorsed by the publisher.
